# Inhibitory Effect of *Chrysanthemum zawadskii* Herbich var. *latilobum* Kitamura Extract on RANKL-Induced Osteoclast Differentiation

**DOI:** 10.1155/2013/509482

**Published:** 2013-09-23

**Authors:** Dong Ryun Gu, Jin-Ki Hwang, Munkhsoyol Erkhembaatar, Kang-Beom Kwon, Min Seuk Kim, Young-Rae Lee, Seoung Hoon Lee

**Affiliations:** ^1^Center for Metabolic Function Regulation (CMFR), Wonkwang University School of Medicine, Iksan 570-749, Republic of Korea; ^2^Department of Oral Microbiology and Immunology, College of Dentistry, Wonkwang University, Iksan 570-749, Republic of Korea; ^3^Department of Oral Biochemistry, College of Dentistry, Wonkwang University, 344-2 Shinyong-dong, Iksan 570-749, Republic of Korea; ^4^Department of Oral Physiology, College of Dentistry, Wonkwang University, Iksan 570-749, Republic of Korea; ^5^Department of Oriental Medical Physiology, College of Korean Medicine, Wonkwang University, Iksan 570-749, Republic of Korea

## Abstract

*Chrysanthemum zawadskii Herbich* var. *latilobum Kitamura*, known as “Gujulcho” in Korea, has been used in traditional medicine to treat various inflammatory diseases, including rheumatoid arthritis. However, these effects have not been tested on osteoclasts, the bone resorbing cells that regulate bone metabolism. Here, we investigated the effects of *C. zawadskii* Herbich var. *latilobum* Kitamura ethanol extract (CZE) on osteoclast differentiation induced by treatment with the receptor activator of NF-**κ**B ligand (RANKL). CZE inhibited osteoclast differentiation and formation in a dose-dependent manner. The inhibitory effect of CZE on osteoclastogenesis was due to the suppression of ERK activation and the ablation of RANKL-stimulated Ca^2+^-oscillation via the inactivation of PLC**γ**2, followed by the inhibition of CREB activation. These inhibitory effects of CZE resulted in a significant repression of c-Fos expression and a subsequent reduction of NFATc1, a key transcription factor for osteoclast differentiation, fusion, and activation *in vitro* and *in vivo*. These results indicate that CZE negatively regulates osteoclast differentiation and may be a therapeutic candidate for the treatment of various bone diseases, such as postmenopausal osteoporosis, rheumatoid arthritis, and periodontitis.

## 1. Introduction

Bone remodeling and metabolism are maintained by a sophisticated regulation between osteoblasts, bone matrix-forming cells, and osteoclasts, bone-resorbing cells [1, 2]. Imbalance between these cells is implicated in the development of bone diseases accompanied by low bone mineral density and bone destruction, such as postmenopausal osteoporosis, periodontitis, and rheumatoid arthritis (RA), which are caused by excessive differentiation and activation of osteoclasts [3–5]. 

Osteoclasts are differentiated from hematopoietic macrophage/monocyte lineage precursor cells in several steps, including proliferation, differentiation, fusion, and activation [2]. Together with the macrophage colony-stimulating factor (M-CSF), receptor activator of NF-*κ*B ligand (RANKL), which is mainly produced by osteoblasts, has been established as a pivotal osteoclast differentiation factor [6, 7]. In RANKL-stimulated osteoclastogenesis, a signal of RANKL binding to its receptor molecules, receptor activator of nuclear factor NF-*κ*B (RANK) expressed on osteoclasts, is transduced into intercellular molecules through TRAF6 adaptor molecule. Thereafter, the c-Jun N-terminal kinase (JNK), extracellular signal-regulated kinase (ERK), p38, Akt, and NF-*κ*B are activated via RANKL/RANK interaction [1]. Subsequent upregulation of c-Fos expression, a positive modulator of osteoclast differentiation, is followed by c-Fos binding to the *NFATc1* promoter region, which induces NFATc1 expression, a master key transcription factor for osteoclastogenesis [8–10]. In addition, RANKL/RANK interaction activates immunoreceptor tyrosine-based activation motif (ITAM) bearing adaptor molecules, such as DNAX-activating protein 12 (DAP12) and Fc receptor common *γ* subunit (FcR*γ*), followed by activation of phospholipase C-gamma (PLC*γ*) [11]. Activation of PLC*γ* leads to the generation of inositol 1,4,5-trisphosphate (IP_3_) and diacylglycerol (DAG) from phosphatidylinositol 4,5-bisphosphate (PIP_2_). Binding of IP_3_ to inositol trisphosphate receptors (IP_3_Rs) on the endoplasmic reticulum (ER) membrane mobilizes Ca^2+^ from the ER stores to the cytosol, causing Ca2^+^-oscillation, which is important for osteoclast differentiation [12, 13]. Ca^2+^-oscillation induces the activation of Ca^2+^/calmodulin-dependent protein kinases (CaMK)IV and cAMP responsive element binding protein (CREB), subsequently leading to induced c-Fos and NFATc1 expression [14]. This CaMKIV/CREB/NFATc1 pathway is also critical to osteoclast differentiation and function [15]. 

*Chrysanthemum zawadskii* Herbich var. *latilobum* Kitamura (Compositae), colloquially known as “Gujulcho” in Korea, has been used in traditional medicine for the treatment of various diseases, including cough, common cold, bladder-related disorders, gastroenteric disorders, hypertension, and inflammatory diseases, such as pneumonia, bronchitis, pharyngitis, and rheumatoid arthritis (RA) [16, 17]. *C. zawadskii* Herbich var. *latilobum* Kitamura extract (CZE) has been shown to harbor many pharmacological properties, including anticancer, antiallergic, anti-inflammatory, and antioxidative stress activities, along with protective effects against liver damage [17–22]. 

Many previously published studies indicate that inflammatory cytokines, including TNF-*α*, IL-1, IL-17, IFN-*γ*, and IL-4, which are produced during successful T-cell-based immune responses, directly regulate RANKL expression on osteoblasts as well as osteoclastogenesis and that inflammation affects bone metabolism [1, 3]. Although CZE has an anti-inflammatory activity, the effect of CZE on bone metabolism has rarely been reported, with the exception that linarin, a component of CZE, prevents hydrogen peroxide-induced dysfunction in osteoblastic MC3T3-E1 cells [23]. However, its effect on osteoclasts still remains unclear. 

 In this study, we investigated the inhibitory effect of CZE on osteoclastogenesis and provided basic mechanisms and possibilities for the use of CZE as a traditional remedy against bone diseases, including osteoporosis, RA, and periodontitis.

## 2. Materials and Methods

### 2.1. Experimental Animals

C57BL/6J (Orient Bio Inc., SeungNam, Korea) were used to generate osteoclasts and for all other experiments. All mouse studies were performed using protocols approved by the Animal Care and Use Committee of Wonkwang University. 

### 2.2. Reagents

The 95% ethanol CZE was purchased from Korean Plant Extract Bank (Daejeon, Korea). All cell culture media, fetal bovine serum (FBS), and supplements were purchased from Hyclone (Rockford, IL, USA). Soluble recombinant mouse RANKL was purified from insect cells as described previously [24], and recombinant human M-CSF was supplied by T Kim (KIOM, Daejeon, Korea). Antibodies against p-ERK, p-JNK, p-p38, p-I*κ*B*α*, p-PLC*γ*2, p-CREB, ERK, JNK, p38, I*κ*B*α*, PLC*γ*2, and CREB were purchased from Cell Signaling Technology (Danvers, MA, USA). Anti-NFATc1 and anti-c-Fos antibodies were purchased from Santa Cruz Biotechnology (Dallas, Texas, USA). 

### 2.3. Cell Viability Assay

Cell viability assays were performed using the EZ-Cytox Enhanced Cell Viability Assay Kit, (Itsbio, Korea), following the manufacturer's instructions. Briefly, bone-marrow-derived macrophages (BMMs), which act as osteoclast precursors, were plated in 96-well culture plates at a density of 1 × 10^4^ cells per well with various concentrations of CZE (0, 2, 5, 10, 25, and 50 *μ*g/mL) for 1 day, or they were cultured with 25 *μ*g/mL of CZE under M-CSF treatment for 4 days. Cells were incubated with EZ-Cytox reagent for 4 h at 37°C. After incubation, the optical density was measured using an ELISA reader (Sunrise, Tecan, Switzerland) at 450 nm. 

### 2.4. *In Vitro* Osteoclast Differentiation

Murine osteoclasts were prepared from bone marrow cells (BM) as previously described [25]. BMs were collected from the tibiae and femora of 6–8-week-old mice by flushing the marrow space with phosphate-buffered saline (PBS). BMs were cultured with M-CSF (30 ng/mL) for 3 days in *α*-minimal essential medium (*α*-MEM) containing 10% FBS, and attached cells were harvested and used as osteoclasts precursors (BMMs). To generate osteoclasts, BMMs were cultured with M-CSF (50 ng/mL) and RANKL (100 ng/mL) for 4 days. Fresh *α*-MEM containing M-CSF and RANKL was replaced on day 3. Cells were fixed with 10% formalin and stained for TRAP. TRAP positive-multinuclear cells (TRAP^+^ MNCs) containing more than three nuclei were counted as osteoclasts. In some experiments, total TRAP activity using *p*-nitrophenyl phosphate (Sigma, USA) as a substrate was measured at an absorbance of 405 nm as previously described [25]. 

### 2.5. Real-Time Quantitative PCR

BMMs treated with or without CZE were cultured with M-CSF (30 ng/mL) and RANKL (100 ng/mL) for 4 days as described above. Total RNA was extracted from cultured cells by using Trizol reagent (Invitrogen, USA) on the indicated days. Then, 1 *μ*g of the total RNA was transcribed to first strand cDNA with random primers using Maxima Reverse Transcriptase (Thermo Scientific, IL, USA) according to the protocol provided by the supplier. Real-time PCR was performed using the VeriQuest SYBR Green qPCR Master Mix (Affymetrix, USA) and StepOnePlus Real-Time PCR Systems (Applied Biosystems, USA). To control for variation in mRNA concentrations, all results were normalized to the GAPDH housekeeping gene. Relative quantitation was performed using the comparative ΔΔ*C*_*t*_ method, according to the manufacturer's instructions. Primers used in this study are listed in Table 1.

### 2.6. Western Blot Analysis

BMMs were cultured with M-CSF (50 ng/mL) and RANKL (100 ng/mL) in the presence or absence of CZE for the indicated time. The cells were washed with cold PBS and lysed in 100 ul of radioimmunoprecipitation assay (RIPA) buffer (25 mM Tris-HCl, pH 7.6, 150 mM NaCl, 1% NP-40, 1% sodium deoxycholate, 0.1% SDS) contained with 1 mM phenylmethylsulfonyl fluoride (PMSF), protease-inhibitor cocktail (Roche, Germany), and phosphatase inhibitor tablets (Thermo Scientific, USA). The cell lysates were cleared by centrifugation at 14,000 ×g for 10 min at 4°C, and the supernatants were collected for immunoblotting. Total lysates (30 *μ*g) were subjected to sodium dodecyl sulfate-polyacrylamide gel electrophoresis (SDS-PAGE) and then transferred to PVDF membranes (Amersham Hybond-P, GE-Healthcare Life Science, USA). Each membrane was blocked for 2 h with 5% skim milk in TBST (TBS; 50 mM Tris-HCl, pH 7.6, 150 mM NaCl, and 0.1% Tween-20) and then incubated with the 1 : 1000 dilution of the primary antibody. HRP-conjugated IgG (1 : 5000 dilutions) was used as the secondary antibody. The immunoreactive proteins were detected using enhanced chemiluminescence (ECL) detection system (Thermo Scientific, USA), according to the manufacturer's protocols. The bands detected were quantitated with the NIH imaging program (NIH Image 1.62), as previously described [26].

### 2.7. Measurement of Ca^2+^-Oscillation

Ca^2+^-oscillation in osteoclasts by RANKL stimulation was measured as previously described with minor modification [27]. BMMs were cultured on the cover slips with RANKL in the presence or absence of CZE (25 *μ*g/mL). After 24 h of RANKL stimulation, intracellular Ca^2+^ mobilization was measured using the fluorescence Ca^2+^ indicator, Fura-2-acetoxymethyl ester (Fura-2AM, 5 *μ*M; TEFLabs, USA). In some cases, BMMs were cultured with RANKL in the absence of CZE for 1 day, and then treated with CZE to verify the acute effects of CZE at the indicated times. Cells were loaded with Fura-2AM for 50 min at room temperature and placed on a chamber connected with a perfusion system. Unloaded fluorescent dye was washed out with bath solution (10 mM HEPES, pH 7.4, 140 mM NaCl, 5 mM KCl, 1 mM MgCl_2_, 1 mM CaCl_2_, and 10 mM glucose; 310 milliosmole). With continuous perfusion of bath solution (37°C), the intracellular fluorescence intensity was measured using two excitation wavelengths (340 and 380 nm), and the emitted fluorescence (510 nm) was captured using a CCD camera. Collected images were digitized and analyzed by MetaFluor software (Ratio = F340/F380). 

### 2.8. Statistical Analysis

Data were analyzed using the Student's two-tailed *t*-test and are presented as mean ± SD values, as indicated. A *P* value of < 0.05 was considered statistically significant. All experiments were repeated at least twice and representative data are shown.

## 3. Results

### 3.1. Effect of CZE on Cell Viability

**Figure 1:**
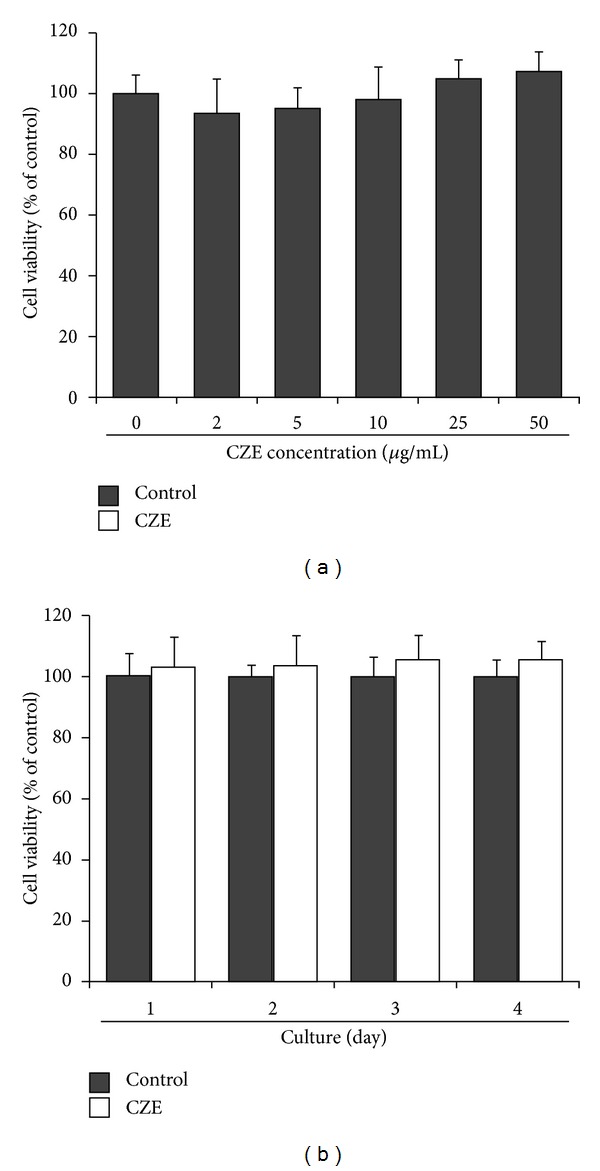
Effects of *Chrysanthemum zawadskii* Herbich var. *latilobum* Kitamura extract (CZE) on cell viability. (a) BMMs were cultured with indicated concentrations of CZE for 1 day. (b) BMMs were cultured with 25 *μ*g/mL CZE or without (control) for 4 days. Cell viability was measured as described in the materials and methods. Data are the mean of three independent experiments (± SD).

To assess the cytotoxicity of CZE on osteoclast precursors, BMMs were treated with various concentrations of CZE (0, 2, 5, 10, 25, or 50 *μ*g/mL) for 1 day. Various concentrations of CZE, up to 50 *μ*g/mL, did not affect the viability of BMMs (Figure 1(a)). All tested concentrations of CZE were shown to have viability levels comparable with that of control. In addition, BMMs were treated with 25 *μ*g/mL of CZE for 4 days. Cell viability was measured daily. There was no significant difference of viability between the control and CZE (25 *μ*g/mL)-treated cells during the 4 days of culture (Figure 1(b)).

### 3.2. Inhibitory Effect of CZE on Osteoclast Differentiation

**Figure 2:**
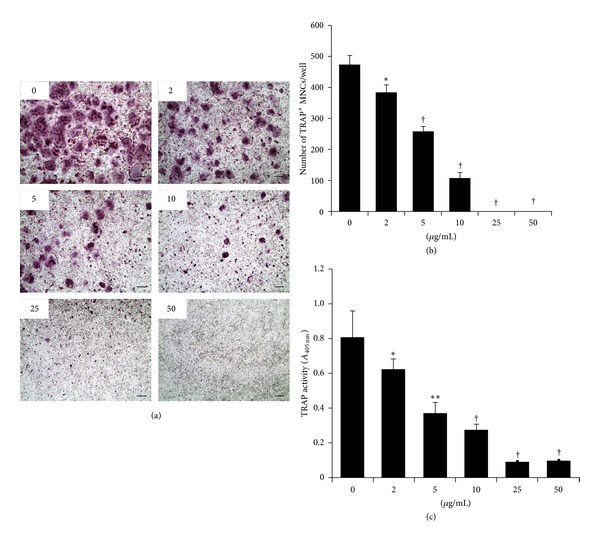
Effects of CZE on osteoclast differentiation. BMMs were cultured with various concentrations of CZE under RANKL and M-CSF treatment for 4 days. (a) Osteoclasts were stained for TRAP. (b) TRAP^+^ multinuclear cells (MNCs) with more than 3 nuclei were counted as mature osteoclasts. (c) Total TRAP activity from TRAP^+^-mono-, di- and multinuclear cells was measured as described in the materials and methods. Data are expressed as the mean ± SD and are representative of at least three independent experiments. *P < 0.05, **P < 0.01, and ^†^ *P* < 0.001 versus control (0 *μ*g/mL CZE). Scale bar = 200 *μ*m.

 To investigate the effect of CZE on osteoclast differentiation, BMMs were cultured with various concentrations of CZE under RANKL treatment for 4 days. Osteoclast differentiation was measured by TRAP staining and TRAP solution assay as previously described. TRAP^+^ MNCs containing more than 3 nuclei and bigger than 100 *μ*m in diameter were counted as mature osteoclasts. CZE treatment dramatically inhibited the formation of mature osteoclasts from BMMs in a dose-dependent manner (Figures 2(a) and 2(b)). When the CZE exceeded 25 *μ*g/mL, mature osteoclasts were rarely formed. In addition, total TRAP activity from mono-, di- and multinuclear osteoclasts was significantly decreased as the CZE concentration increased (Figure 2(c)). These data suggest that the role of CZE is to repress osteoclast differentiation from the precursor cells to mature osteoclasts, and that it is not involved with the osteoclast fusion and activation steps.

**Figure 3:**
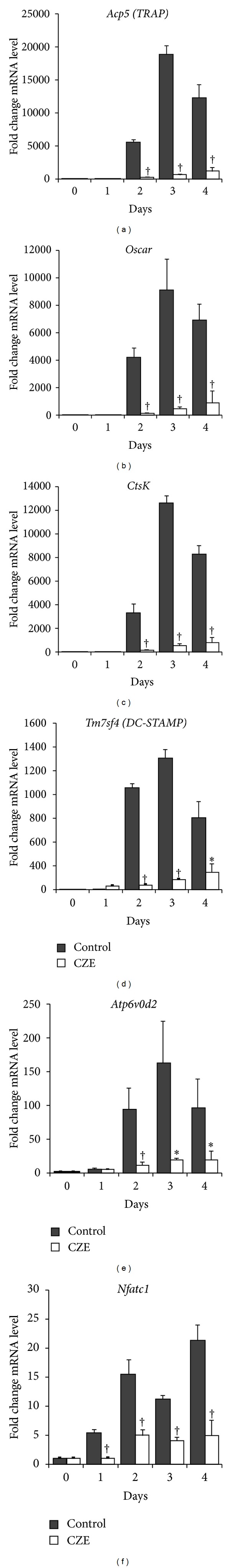
Effects of CZE on the expression of osteoclast differentiation marker genes. BMMs were cultured with RANKL and M-CSF treatment in the presence or absence of CZE (25 *μ*g/mL) for 4 days. The expression of osteoclast differentiation marker genes was measured by real-time PCR. The expression of mRNA levels was normalized with GAPDH and described as fold change of mRNA level. Data are expressed as the mean ± SD and are representative of at least three independent experiments. *P < 0.05, and ^†^ *P* < 0.01 versus control (0 *μ*g/mL CZE).

 To confirm the inhibitory effect of CZE on osteoclast differentiation, the expression of osteoclast differentiation marker genes (*Acp5, Oscar, CtsK, Tm7sf4,* and *Atp6v0d2*) and a master transcription factor for osteoclast differentiation, *Nfatc1*, were measured during RANKL-induced osteoclast differentiation. As shown in Figure 3, CZE significantly inhibited the expression of all tested marker genes and *Nfatc1*. Together with Figure 1, these results indicate that CZE has an inhibitory effect on RANKL-induced osteoclast differentiation and formation without cytotoxicity.

### 3.3. Suppression of c-Fos and NFATc1 Expression via ERK Inactivation by CZE

**Figure 4:**
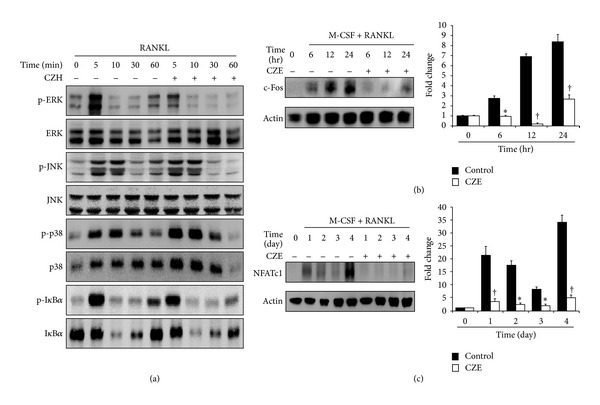
Effects of CZE on RANKL-induced intracellular signaling and the expression of transcription factors in osteoclasts. BMMs were treated with M-CSF and RANKL in the presence or absence of CZE (25 *μ*g/mL) for the indicated time. Lysate (30 *μ*g) was subjected to SDS-PAGE and analyzed by immunoblotting. (a) MAPK (ERK, JNK, and p38) activation was measured by using their respective antibodies. (b)-(c) The expression of c-Fos and NFATc1 was detected by anti-c-Fos and NFATc1 antibody, respectively. Fold change normalized by actin is presented in the right panel. Data are representatively obtained from three independent experiments and are expressed as the mean ± SD. *P < 0.05, and ^†^ *P* < 0.01 versus control (0 *μ*g/mL CZE).

In RANKL-induced osteoclast differentiation, RANKL/RANK signaling induces the activation of NF-*κ*B and mitogen-activated protein kinases (MAPKs), followed by c-Fos expression, which results in the induction of NFATc1, a key transcription factor for osteoclastogenesis [1]. Therefore, we investigated the effect of CZE on the regulation of RANKL-induced signaling pathways. First, the BMMs were treated with or without CZE (25 *μ*g/mL) under RANKL and M-CSF treatment, and then, the activation of MAPKs and I*κ*B*α* was measured by western blot analysis. As shown in Figure 4(a), the phosphorylation of ERK diminished in the CZE-treated cells compared to that of control cells. However, the activation of I*κ*B*α* and other MAPKs (JNK and p38) was not significantly changed. Next, we measured the expression levels of c-Fos and NFATc1. When CZE was treated, the expression of c-Fos was dramatically repressed 6 h after treatment in osteoclast differentiation (Figure 4(b)). In addition, the induction of NFATc1 was significantly inhibited by CZE treatment (Figure 4(c)), coinciding with mRNA expression patterns (Figure 3). These results indicate that CZE inhibited the expression of c-Fos via the inactivation of ERK in RANKL-induced osteoclast differentiation. This inactivation leads to the repression of the expression of NFATc1, which regulates all the steps involved in osteoclast differentiation, fusion, and activation. 

### 3.4. Breakdown of Intracellular Ca^2+^-Oscillation and Inhibition of PLC*γ*2 and CREB Activation by CZE

**Figure 5:**
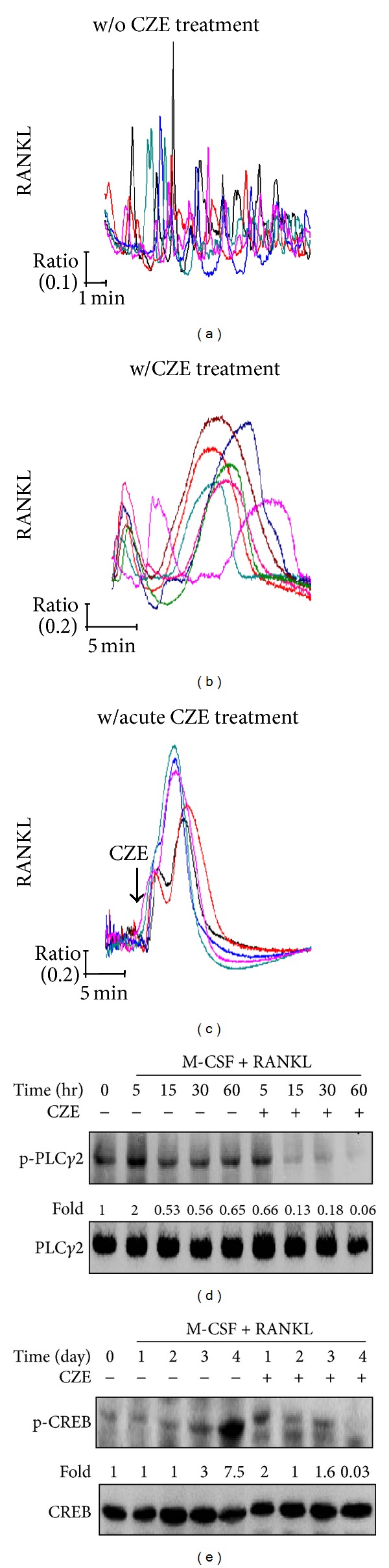
Effect of CZE in RANKL-stimulated costimulatory signals in osteoclasts. BMMs were cultured with M-CSF and RANKL in the presence or absence of CZE (25 *μ*g/mL) for 1 day. (a)-(b) RANKL-induced Ca^2+^-oscillation was measured with the Ca^2+^ indicator, Fura-2AM. (c) BMMs were cultured with M-CSF and RANKL in the presence of CZE for 1 day. RANKL-induced Ca^2+^-oscillation by acute treatment of CZE was measured. (d)-(e) BMMs were cultured with M-CSF and RANKL in presence or absence of CZE (25 *μ*g/mL) for the indicated time. Lysate (30 *μ*g) was subjected to SDS-PAGE, and the activation of PLC*γ*2 (d) and CREB (e) was analyzed by immunoblotting. All data are representative of at least three independent experiments.

**Figure 6:**
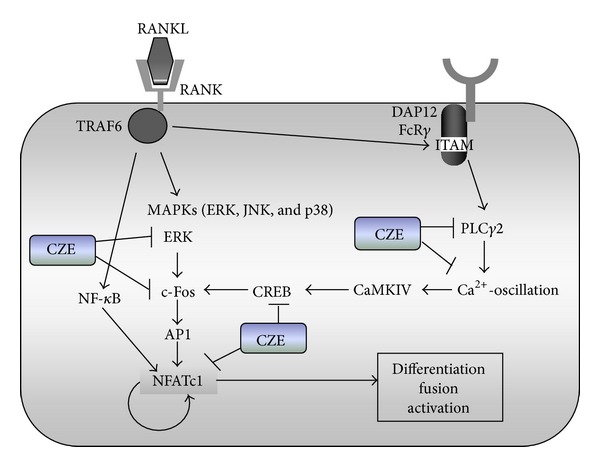
Schematic diagram of the effect of CZE on RANKL-induced osteoclastogenesis. RANKL/RANK interaction may lead to the activation of MAPKs followed by c-Fos expression and alternatively activation of PLC*γ*2 inducing calcium signaling, which is critical for NFATc1 activation, followed by CREB activation and induction of c-Fos and NFATc1. CZE inhibited both RANKL-induced ERK and PLC*γ* activation signaling pathways.

 In addition to MAPK and NF-*κ*B activation, RANKL/RANK signaling also activate phospholipase C gamma 2 (PLC*γ*2) and induces Ca^2+^-oscillation, followed by CREB activation [14, 25, 28]. CREB is critical for RANKL-stimulated NFATc1 and c-Fos induction in osteoclast precursors [14]. We first examined whether CZE affects the induction of Ca^2+^-oscillation by RANKL stimulation. BMMs were cultured with or without CZE under RANKL stimulation for 24 h. Intracellular Ca^2+^ concentration was measured as described previously. Control cells exhibited typical Ca^2+^-oscillation as shown in Figure 5(a). However, CZE-treated cells showed an irregular Ca^2+^-oscillation pattern with significantly increased intensity, but without increased frequency (Figure 5(b)). In addition, we acutely added CZE on control cells showing typical Ca^2+^-oscillation and then measured Ca^2+^ mobilization. As shown in Figure 5(c), Ca^2+^-oscillation was defective in these cells, with a large Ca^2+^ influx peak after CZE treatment. It seems that CZE may interact with some Ca^2+^ channels, which contributes to a substantial Ca^2+^ influx into the cells. We next examined whether RANKL-stimulated PLC*γ*2 activation is affected by CZE treatment. Phosphorylation of PLC*γ*2 in CZE-treated cells was significantly inhibited. In addition, the activation of CREB by RANKL was dramatically suppressed during osteoclast differentiation in CZE-treated cells. Collectively, these data demonstrate that CZE regulates not only MAPKs and NF-*κ*B activation, but also PLC*γ*2 activation and RANKL-induced Ca^2+^-oscillation, which are important for CREB activation and c-Fos and NFATc1 induction in RANKL-stimulated osteoclast differentiation (Figure 6). 

## 4. Discussion

Although *C. zawadskii* Herbich var. *latilobum* Kitamura has routinely been used as a traditional remedy against several inflammatory diseases and the mechanisms for its anti-inflammatory effects have been studied [16, 17], comparatively little is known about its effect against inflammation-related bone diseases such as RA and periodontitis or on bone cells (osteoclasts and osteoblasts). Only the effect of linarin on osteoblastic MC3T3 cells has been reported that it inhibits cytotoxicity and oxidative damage, and restores the mineralization function of hydrogen peroxide-treated osteoblasts. Linarin also suppresses RANKL expression induced by hydrogen peroxide and appears to have antiresorptive activity [23]. Here, we have elucidated an inhibitory effect of CZE via the reduction of NFATc1 expression in the differentiation and formation of osteoclasts, which cause bone destruction associated with inflammation-related bone diseases.

Previously, many studies have established that NFATc1 is a critical transcription factor for RANKL-mediated osteoclast differentiation, fusion, and activation. When BMMs are stimulated by RANKL, the expression of NFATc1 is induced through c-Fos and autoamplification by NFATc1 [9, 10]. NFATc1-deficient embryonic stem cells do not form mature osteoclasts by RANKL treatment and overexpression of ectopic ca-NFATc1 in BMMs appropriately induces osteoclast differentiation from BMMs even in the absence of RANKL [9, 25, 29]. Recently, we had reported that NFATc1 is a key regulator of osteoclast fusion, which is an essential step for efficient bone resorption, via upregulation of ATP6v0d2 and dendritic cell-specific transmembrane protein (DC-STAMP), which are known as osteoclast fusion molecules as confirmed by genetic experiments [29]. Moreover, several reports showed that NFATc1 is implicated in the regulation of osteoclast function. The expression of TRAP, Cathepsin K, c-Src, and *β*3 integrin, which are involved in osteoclast-mediated bone resorption, is regulated by NFATc1 [9, 10, 12]. Furthermore, acidosis and RANKL signals in osteoclasts stimulate bone resorption via activation of Ca^2+^/calcineurin/NFAT pathway [30]. These results indicate that NFATc1 is a master key regulator of osteoclastogenesis. Therefore, in order to regulate excessive osteoclasts activity, which causes severe bone destruction in bone diseases, it is efficient and essential to control the expression of NFATc1 as a therapeutic target. In this study, our data demonstrate that CZE suppresses the expression of c-Fos and NFATc1 via inactivation of ERK, which contribute to RANKL-induced osteoclast differentiation (Figure 4). 

Previous studies elucidated that Ca^2+^-oscillation for NFATc1 induction is an essential process for osteoclastogenesis, and that ablation of Ca^2+^-oscillation causes impairment of osteoclastogenesis [12, 31, 32]. RANKL-stimulated Ca^2+^-oscillation is initiated approximately 24 h after RANKL treatment and is maintained until the formation of mature osteoclasts [15]. It seems that long-term Ca^2+^-oscillation is helpful for sustaining NFATc1 in the nucleus and for ensuring the transcriptional activation of NFATc1 required for terminal osteoclast differentiation [33]. Intracellular Ca^2+^ originates from the extracellular space through plasma membrane channels or intracellular organelles such as the ER [34]. IP_3_ receptors (IP_3_Rs), especially IP_3_R2 and IP_3_R3 in osteoclasts, mediate Ca^2+^ release from the ER to the cytosol in response to IP_3_ binding [13]. Recently, it has been reported that the Ca^2+^ influx during osteoclastogenesis is regulated by plasma membrane-localized Ca^2+^ channels, such as Orai1, TRPV4, and TRPV5 [35–37]. 

In Figure 5, BMMs treated with RANKL for 1 day produced typical Ca^2+^-oscillation. However, when BMMs were treated with CZE under RANKL stimulation for 1 day, the typical Ca^2+^-oscillation was interrupted; instead, abnormal biphasic pick, which is thought to be mediated by Ca^2+^ influx, was induced. In addition, when RANKL-stimulated BMMs showing typical Ca^2+^-oscillation were acutely treated with CZE, an abnormal biphasic pick, implicating dramatically increased intracellular Ca^2+^ concentration, [Ca^2+^]_*i*_ was produced, and the typical Ca^2+^-oscillation by RANKL disappeared. Although it is unknown how CZE induces abnormal Ca^2+^ influx in osteoclasts, CZE definitely ablated the typical Ca^2+^-oscillation induced by RANKL stimulation. In addition, CZE inhibited PLC*γ*2 and CREB activation, which are important for osteoclast differentiation, followed by the repression of c-Fos and NFATc1 expression. These results indicate that CZE is an effective regulator of osteoclast differentiation and formation via the control of two main pathways induced by RANKL/RANK binding. In further studies, it will be interesting to explore how CZE regulates Ca^2+^ influx and which Ca^2+^ channel is affected by CZE. Detailed investigations of this mechanism will provide more insights into the effect of CZE on osteoclastogenesis and its relationship with the pathologies of bone diseases, such as osteoporosis, periodontitis, and rheumatoid arthritis. 

## 5. Conclusion

Our results clearly demonstrate that the inhibitory effect of CZE on RANKL-stimulated osteoclastogenesis is mediated by the repression of c-Fos and NFATc1 expression, which are critical for osteoclastogenesis, via ERK and PLC*γ*/Ca^2+^-oscillation/CREB signaling in osteoclasts. These findings reveal CZE as a traditional therapeutic agent against inflammatory bone diseases, such as rheumatoid arthritis and periodontitis.

## Figures and Tables

**Table 1:** Nucleotide sequences of the primers used for real-time PCR in this study.

Gene	Primers
*Acp5 (TRAP) *	Forward 5′-CTGGAGTGCACGATGCCAGCGACA-3′
	Reverse 5′-TCCGTGCTCGGCGATGGACCAGA-3′
*Oscar *	Forward 5′-GGGGTAACGGATCAGCTCCCCAGA-3′
	Reverse 5′-CCAAGGAGCCAGAACGTCGAAACT-3′
*Cathepsin K (CtsK) *	Forward 5′-ACGGAGGCATTGACTCTGAAGATG-3′
	Reverse 5′-GTTGTTCTTATTCCGAGCCAAGAG-3′
*Tm7sf4 (DC-STAMP) *	Forward 5′-TGGAAGTTCACTTGAAACTACGTG-3′
	Reverse 5′-CTCGGTTTCCCGTCAGCCTCTCTC-3′
*ATP6v0d2 *	Forward 5′-TCAGATCTCTTCAAGGCTGTGCTG-3′
	Reverse 5′-GTGCCAAATGAGTTCAGAGTGATG-3′
*Nfatc1 *	Forward 5′-CTCGAAAGACAGCACTGGAGCAT-3′
	Reverse 5′-CGGCTGCCTTCCGTCTCATAG-3′
*Gapdh *	Forward 5′-TGCCAGCCTCGTCCCGTAGAC-3′
	Reverse 5′-CCTCACCCCATTTGATGTTAG-3′
